# Gut dysbiosis and microbial metabolites in atopic dermatitis: implications for immune regulation along gut-skin axis

**DOI:** 10.3389/fmicb.2026.1829876

**Published:** 2026-05-15

**Authors:** Khushbakhat Alia, Hira Khan, Hamna Muzaffar, Nighat Perveen, Sara Alrashedi, Yusra Al Dhaheri, Yasir Waheed, Mohammed Tauqeer Alam, Muhammad Naseem, Khalid Muhammad

**Affiliations:** 1 Department of Biology, College of Science, United Arab Emirates University, Al Ain, United Arab Emirates; 2 Széchenyi István University, Győr, Hungary; 3 NUST School of Health Sciences, National University of Science and Technology, Islamabad, Pakistan; 4 College of Natural and Health Sciences, Department of Environmental Sciences and Sustainability, Zayed University, Abu Dhabi, United Arab Emirates; 5 Department of Bioinformatics, Biozentrum Am Hubland, University of Würzburg, Würzburg, Germany; 6 Zayed Bin Sultan Al Nahyan Center for Health Sciences, United Arab Emirates University, Al Ain, United Arab Emirates

**Keywords:** atopic dermatitis, epithelial barrier integrity, gut microbiota dysbiosis, gut-skin axis, immune dysregulation, microbial metabolites

## Abstract

Atopic dermatitis (AD) is a multifactorial skin disorder characterized by immune dysregulation, impaired epidermal barrier, and strong microbial imbalance. Although genetic susceptibility and environmental triggers are established AD drivers, growing evidence highlights the gut skin axis as an important but underexplored AD pathogenesis factor. Gut microbiota dysbiosis, loss of short-chain fatty acid (SCFA)-producing bacteria, and altered metabolite profiles, such as tryptophan derivatives and secondary bile acids, have been linked to systemic immune imbalance and skin inflammation. However, the precise mechanism by which gut microbial alterations influence cutaneous immunity remains unclear. This review synthesizes recent advances from clinical and experimental studies to delineate how the gut microbiota and their metabolites shape the immune response, regulate the integrity of the epithelial barrier, and modulate AD severity. By integrating emerging insights into early-life microbial colonization, metabolite-mediated immune programming, and therapeutic interventions, including prebiotics, probiotics, and microbial-derived metabolites, the current gaps and the translational potential of targeting the gut- skin axis. The knowledge consolidated here advances our understanding of AD beyond skin-focused perspectives and highlights new avenues for microbiome-based preventive and therapeutic strategies.

## 1 Introduction

Atopic dermatitis, also known as eczema, is a heterogeneous, recurrent, and chronic inflammatory skin disease with flares and remissions. It is characterized by itch, pain, and scaly lesions, dry skin, and abnormal pruritus (Ong, 2022; Chen M. et al., 2024; Chen Y. et al., 2024; Criado et al., 2024; Ozdemir and Oksuz, 2024; Zhou et al., 2024; Nagashima et al., 2024; Purevsuren et al., 2024; Mo et al., 2025). Other allergic diseases (allergic rhinitis, asthma, and food allergies) are also associated with AD. Globally, the prevalence of AD is increasing, and its prevalence in children is higher with an onset below the age of 5 years (15–30%) than in adults (2–10%). Environmental factors may increase disease prevalence by affecting genetic predisposition. Common allergens, such as pollen, dust mites, and animals, can trigger the symptoms of AD. Although the cause and mechanism of AD development are complex, with multiple pathogenic mechanisms, the role of immune dysregulation and gut microbiome is important. The immune system and human gut microbiome are co-related in AD development, as some microorganisms in the human body stimulate the immune system development and maturation. Studies have shown that the mode of delivery and feeding methods affect the infant’s gut microbiome composition. Studies have also shown that the structure of the gut microbiome is critical for long-term health in early life (Zhou et al., 2024; Losol et al., 2023; Pessoa et al., 2023; Purevsuren et al., 2024). Certain changes in the gut microbial flora can lead to abnormal immune responses in skin inflammation. High IgE levels and skin Th2 cytokine expression is associated with changes in the gut microbiome in AD. In all skin types, AD is associated with a type-2 inflammatory pattern (Qiuyu et al., 2024; Gan et al., 2023; Zhou et al., 2024), elevated serum IgE, structural defects in skin barrier proteins, and sensitization to allergens (Alam et al., 2022; Purevsuren et al., 2024). In acute AD, disruption of the epidermal barrier stimulates keratinocytes to release cytokines and chemokines to activate dendritic cells and Langerhans cells to present antigens to Th2 cells and cause the release of cytokines that give rise to further barrier dysfunction, itch, and impaired keratinocyte differentiation. Th17, Th22, and Th1 cell activation, epidermal thickening, and abnormal keratinocyte differentiation are associated with chronic AD (Gan et al., 2023).

This review provides a comprehensive analysis of the multifactorial pathogenesis of AD, with a primary focus on how gut microbiota dynamics contribute to disease development and progression, while also considering the interplay between immune dysregulation, epidermal barrier impairment, and gut-skin axis signaling.

## 2 Methodology

This article is a narrative review conducted through a peer-reviewed literature search to provide current evidence on the role of gut microbiota in the pathogenesis of AD. A comprehensive search was performed using the PubMed, Scopus, and Web of Science databases. The following keywords and their combinations were used: “Atopic Dermatitis,” “gut microbiota,” “gut-skin axis,” “Th2 inflammation,” “SCFA,” “microbial metabolites,” “gut microbiota and Atopic Dermatitis,” “SCFA,” “role of SCFAs in atopic dermatitis,” and “immune system modulation in Atopic Dermatitis”. Studies published between 2016 and 2024 were prioritized, with a focus on original research articles, experimental models, and systematic reviews. Earlier studies were also included where relevant. Eligible studies comprised original research articles, clinical studies, *in vivo* murine models, and *in vitro* investigations, and studies exploring the relationship between microbial metabolites and immune regulation were evaluated to synthesize the current understanding of the gut-skin axis in AD pathophysiology.

Articles were screened based on relevance of the title and abstract, followed by text evaluation. Studies included if they directly addressed gut microbiota composition, microbial metabolites, immune system regulation, or gut-skin axis mechanism in AD. The exclusion criteria included non-English publications, conference abstracts without full text, and studies not directly related to AD, the role of gut microbiota in AD, or microbiota-mediated mechanisms.

Studies with robust experimental design, clinical relevance, and mechanistic insights were prioritized. In cases of conflicting findings, reproducibility, study design quality, and consistency across multiple reports were emphasized. This review does not follow a formal systematic review protocol or meta-analysis due to the heterogeneity of study designs, models, and outcome measures in the available literature.

## 3 Immunopathogenesis of AD

The immunopathogenesis of AD is associated with the dysregulation of the immune response through a multidimensional cascade involving the induction of the inflammatory response, disruption of immune homeostasis, architectural disturbance of the epidermal and dermal interface, and the production of pruritus, a cardinal symptom (Criado et al., 2024). In the pathogenesis of AD, the abnormal immune response is associated with the predominance of differentiation of CD4 lymphocytes toward the Th2 lineage (Mazur et al., 2023). A Th1/Th2 immune imbalance is a defining feature of AD.

The dominance of Th2-driven inflammation, elevated expression of type 2 cytokines, Interleukin (IL)-4, IL-13, and IL-5, is central to AD. In AD, keratinocytes release proinflammatory cytokines (TSLP, IL-33, IL-25) upon exposure to irritants. These alarmins activate basophils and innate lymphoid cells 2 (ILC2s) to release IL-4, IL-5, and IL-13. These cytokines amplify the Type 2 inflammatory response (Chen Y. et al., 2024; Ong, 2022; Mazur et al., 2023; Chen M. et al., 2024; Chen Y. et al., 2024; Criado et al., 2024; Zhou et al., 2024). The overproduction of IL-13 and IL-4, followed by Th2 cell activation, promotes the production of antigen-specific immunoglobulins (IgE) by B cells. The subsequent binding of IgE to the FcεRI receptor on basophils and mast cells stimulates cell degranulation and the release of proinflammatory mediators. Concurrently, keratinocytes in AD skin upregulate the expression of epithelial-derived cytokines, including thymic stromal lymphopoietin (TSLP), IL-33, and IL-25, which are responsible for initiating itch and scratching. These proinflammatory cytokines also cause differentiation of naïve T cells into Th2 cells or through the involvement of dendritic cells. Activated Th2 cells release IL-31, which promotes itch and scratching and damages the epidermal barrier (Mazur et al., 2023; Chen Y. et al., 2024). The activation of Th2 immune responses by epidermal damage and exposure to allergens deregulates Tregs, thereby sustaining the inflammatory response (Criado et al., 2024).

**Figure 1:**
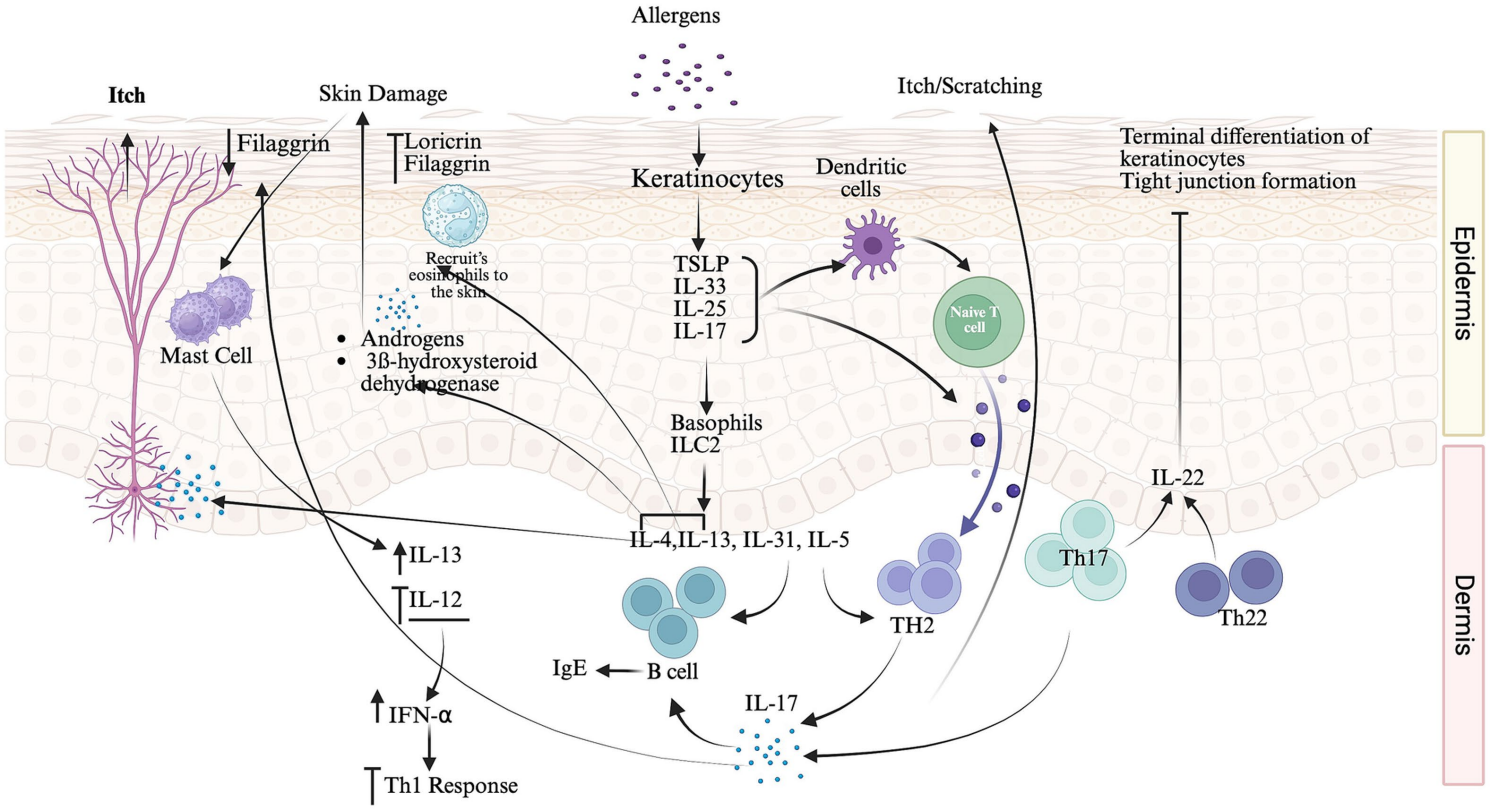
Mechanisms of allergen-induced skin itching and inflammation. Schematic representation of the key cellular and molecular pathways involved in allergen-induced pruritus and cutaneous immune responses. Skin barrier disruption associated with reduced filaggrin or loricrin levels leads to dendritic cell activation by keratinocytes. Mast cells and basophils release pruritogenic cytokines (IL-4, IL-13, and IL-31) and promote IgE-mediated B-cell responses, whereas ILC2s amplify type 2 immunity. Additionally, Th1 (IFN-*γ*), Th17 (IL-22), and Th22 pathways contribute to dermal inflammation and itch-scratch cycles.

Acute AD is predominantly driven by a type 2 response, triggered by inflammasomes, alarmins, and epidermal and dermal antigen-presenting cells, whereas Th1 and Th17 cells became more active in chronic AD. In the chronic phase, Th22 by releasing IL22, and Th17 cells play a role in AD development by inhibiting the terminal differentiation of keratinocytes and tight junction formation and contributing to the disruption of the epidermal barrier (Figure 1). IL-22 increases antigen sensitization and contributes to pruritus pathogenesis In the chronic phase, tissue remodeling and IL-17 release from eosinophils increase the production of pro-fibrotic factors, such as IL-13 and TGF-*β*. IL-17 plays a role in the differentiation of naive B cells and IgE production (Chen Y. et al., 2024; Chen M. et al., 2024; Criado et al., 2024) and causes the downregulation of the expression of FLG, a protein responsible for maintaining the epidermal barrier. In comparison with normal skin, the lesional skin of patients with AD had higher levels of IL-9 and IL-10. The role of IL-10 in the production of antimicrobial peptides in AD patients is ambiguous, but some studies have indicated an inverse correlation between IL-10 levels and disease severity (Ozdemir and Oksuz, 2024).

## 4 The gut–skin axis in AD

The skin is the largest organ of the human body and acts as a primary barrier to protect the body’s interior from microbial invasion and external threats. Recent research highlights the importance of the “gut–skin axis,” which refers to a bidirectional communication network between the gut microbiota and the microbial and immune environment of the skin. The gut–skin axis is crucial in AD. Although the precise mechanisms are not fully understood, an imbalanced microbiota is believed to play a significant role in the inflammation and immunological responses associated with AD. Gut dysbiosis has been linked to various dermatological conditions, including AD, acne, psoriasis, wrinkles, and aging (Pessoa et al., 2023; Xiao et al., 2023; Salvadori and Rosso, 2024).

AD is also associated with skin microecology imbalance. The reduced abundance of probiotic bacteria in the skin of patients with AD promotes the growth and colonization of pathogenic bacteria (Xiao et al., 2023), which intensifies skin inflammation. In the skin of 60–100% of patients with AD, colonization contained *Staphylococcus aureus* compared with 5–30% of healthy controls. Deformed corneocytes, decreased levels of filaggrin, deficiency of AMPs, overexpression of Th2 inflammatory cytokines, and altered lipid profiles contribute to the increased colonization of *S. aureus* in the skin (Li et al., 2021). The increased colonization by *S. aureus* in the skin also results in increased expression of several antigens and virulence factors, such as cytolytic a, d-toxins, PSMs, protein A, and several proteases, which can awaken the adaptive immunity of the dermis in the EIME. Several studies on EIME have demonstrated the complexity of AD triggered by adaptive immunity and influenced by abnormal microbes (Wu et al., 2025).

In the skin microbiome of patients with AD, the loss of the anaerobic bacteria *Faecalibacterium prausnitzii* results in a disturbed gut microbiome, which results in the dysregulation of the gut epithelium and inflammation (Purevsuren et al., 2024). Predominant gram-positive and facultative anaerobic bacteria (Firmicutes, Proteobacteria, Actinobacteria, and Bacteroidetes) in the skin ecosystem play critical roles in immune regulation and pathogen defense. For skin health and integrity, the interaction between bacteria from genera such as *Staphylococcus*, *Propionibacterium, Streptococcus*, and *Corynebacterium* is important. An imbalance in the gut microbiome disrupts mucosal immune tolerance and affects skin health, indicating that gut microbiota dysbiosis is linked with skin health and diseases (Chen M. et al., 2024).

One proposed mechanism for the development of AD is molecular mimicry occurring between environmental allergens (pollen or dust mites) and self-antigens. Allergens, dust mites, and pollen share structural similarities with profilaggrin and other skin proteins. These allergens may elicit an immune response against both the external allergen and the self-antigen upon contact with the skin, resulting in inflammation and tissue damage (Pessoa et al., 2023).

Gut microbiota dysbiosis disrupts short-chain fatty acid (SCFA) production, which is essential for maintaining intestinal barrier integrity and immune homeostasis. Reduced SCFA levels may increase intestinal permeability, facilitating microbial antigen translocation into the systemic circulation. They can be processed by antigen-presenting cells and activate cross-reactive T cells. This process contributes to immune tolerance breakdown and promotes autoreactive responses against structurally similar self-antigens (Czaja, 2023).

Patients with AD exhibit an increased proportion of *Clostridium difficile*, *Escherichia coli*, and *S. aureus* and a decreased proportion of beneficial microbes such as *Lactobacillus*, *Bifidobacteria,* and *Bacteroides* (Zhou et al., 2024; Lee et al., 2018; Jung Eun and Hei Sung, 2019; Fang et al., 2022) compared with healthy controls. Healthy individuals typically harbor dominant phyla such as Bacteroidetes, Actinobacteria, Firmicutes, and Proteobacteria. Patients with severe AD have a smaller number of *Coprococcus eutactus* (butyrate-producing bacteria) *Bifidobacterium, Blautia, Eubacterium,* and *Propionibacterium* than healthy individuals (Lee et al., 2018). The severity of AD correlates with reduced levels of SCFA-producing bacteria, depending on the severity of the disease. Individuals with acute AD have lower levels of *Bifidobacteria* than those with moderate atopy (Ozdemir and Oksuz, 2024). Supplementation of *AD patients with Bifidobacteria* increases the level of tryptophan metabolite in feces, which improves itching symptoms and colonic mucosal function (Chen M. et al., 2024).

## 5 Early-life colonization: immune programming and susceptibility to AD

Gut microbial colonization plays a fundamental role in immune development and AD susceptibility during infancy and early childhood. Gut microbiota plays a pivotal role in shaping the host immune system, and gut microbial dysbiosis has been strongly associated with immune-mediated disorders. Changes in the gut microbiome affect the immune system balance by producing certain metabolites and inflaming the microenvironment. Establishing a diverse and stable microbiota is critical for immune maturation in early life. Microbial exposure during this developmental window helps shape T cell repertoires and regulatory pathways that determine immune setpoints later in life (Lee et al., 2018; Fang et al., 2021; Zhou et al., 2024).

According to the “hygiene hypothesis,” reduced microbial exposure during early life impairs immune tolerance, resulting in an increased prevalence of allergic diseases, including AD (Zhu et al., 2019; Qiuyu et al., 2024). During pregnancy, changes in the maternal gut microbiota can affect the immunity of the offspring in the early postnatal period (Fang et al., 2021). Within the first 3 years of life, intestinal microbe colonization gradually stabilizes and assumes an adult-like profile, a process critical for immune maturation (Pessoa et al., 2023). Evidence from germ-free mouse models indicates that the absence of gut microbiota results in profound immune dysfunction. Epidemiological studies have demonstrated that infants with higher gut microbial diversity exhibit a low risk of developing AD at the age of 1 week. Conversely, a decreased diversity of gut microbiota and delayed colonization of Bacteroidetes at 1 month of age have been associated with a high risk of AD development (Jung Eun and Hei Sung, 2019). Infants with AD frequently exhibit reduced abundances of beneficial bacteria such as *Bifidobacterium, Bacteroides*, *Akkermansia*, and *Faecalibacterium*. In contrast to these species, infants with AD have a higher population of *Staphylococcus*, *Enterobacteriaceae*, *Candida*, *Rhodotorula*, and *Clostridium* species. These compositional shifts in early life, particularly during weaning, are crucial for the optimal development of the immune system (Qiuyu et al., 2024). Infants born by cesarean section are more likely to develop AD compared to those born by vaginal delivery. The gut microbiota tends to be enriched in genera such as *Roseburia*, *Clostridium*, and *Anaerostipes* after breastfeeding cessation (Jung Eun and Hei Sung, 2019; Fang et al., 2021). The role of the maternal microbiota in shaping neonatal immunity is supported by experimental studies. For instance, in gestation, the transient colonization of female mice with *Escherichia coli* HA107 altered the numbers of intestinal leukocytes in offspring and affected the development of the early innate immune system (Gomez de Agüero et al., 2016; Fang et al., 2021). Additionally, Melli et al. (2020) reported that children (5–11 years old) with AD have a higher population of *Bifidobacterium* and *Clostridium* and a decreased population of *Lactobacillus* (Melli et al., 2020). Microbial diversity in the gut remains a critical determinant of AD risk. A cohort study involving 1,440 children aged 10 years revealed a strong association between higher *α* diversity of gut microbiota and reduced incidence of eczema (Hu et al., 2021). The concept “microbial deprivation syndromes of affluence” explains that reduced intensity and diversity of microbial exposure during early life impairs Th1 induction, thereby creating a predisposition toward Th2-skewed immune responses. This immunological imbalance triggered by dysbiotic gut microbiota, gut inflammation, and a disrupted epithelial barrier may contribute to the pathogenesis of AD.

Colonization of *S. aureus* in the gut of infants and particulars strains harboring super-antigen and adhesin genes has been associated with increased AD risk. Regulation of the number of gut *Bifidobacterium* regulates the expression of IL-10, which plays a role in the development of AD (Zhou et al., 2024). The presence of *Clostridia* and *Escherichia* in the gut may also contribute to eosinophilic inflammation, further linking intestinal dysbiosis to AD pathophysiology (Lee et al., 2018).

Different microbial signatures were observed in children with and without AD and food allergies. The fecal samples from children with AD and food allergies exhibited high levels of *B. pseudocatenulatum* and *E. coli and* decreased levels of *B. adolescentis*, *B. breve, F. prausnitzii, and A. muciniphila* (Pessoa et al., 2023; Ozdemir and Oksuz, 2024). A study reported that the presence of *A. muciniphila* and *R. gnavus* in the gut microbiome is associated with alterations in immune-regulatory gene expression and affects the overall development of the host immune system. However, there is no consensus that the compositional changes in the gut microbiome precede the development of immune system dysregulation and epithelial barrier impairment in AD or that the established gut microbiome plays a role in the onset of AD (Lee et al., 2018). Specific Clostridia strains have been shown to promote the expansion and differentiation of regulatory T cells (Tregs) in murine models, alleviating the clinical symptoms of allergic diarrhea and colitis (Atarashi et al., 2013; Fang et al., 2021). Similarly, segmented filamentous bacteria residing in the small intestinal lamina propria induce Th17 cell differentiation and contribute to the development of autoimmune arthritis (Fang et al., 2021). Furthermore, the loss of microbial-driven immune tolerance is increasingly recognized as a key mechanism underlying the development of autoimmune and allergic diseases (Salvadori and Rosso, 2024). Consistent alterations in gut microbiota composition in AD have been reported, characterized by shifts in major bacterial phyla and key commensal genera, as summarized in Table 1.

**Table 1:** Representative microbial shifts observed in AD.

Study type	Main taxa	Observed effects	Principal limitations	References
Case control study in patients with AD and healthy controls	Lower *Bifidobacterium* and higher *Staphylococcus* in AD	Suggested altered gut microbial balance in AD and possible relevance to disease severity	Cross sectional design, older culture based methods and small sample size.	Watanabe et al. (2003)
Gut microbiome study in patients with AD and controls	*Faecalibacterium prausnitzii* dysbiosis with lower fecal butyrate and propionate	Linked microbial and metabolite changes to epithelial dysfunction and immune imbalance relevant to AD	Spports association and mechanistic plausibility but does not establish causality between gut dysbiosis and AD	Song et al. (2016)
Cross sectional pediatric AD study with food allergy stratification	Higher *Escherichia coli* and *Bifidobacterium pseudocatenulatum*. Lower *Bifidobacterium breve, Bifidobacterium adolescentis, Faecalibacterium prausnitzii* and *Akkermansia muciniphila* in food allergic AD subgroup	Altered immune tolerance with potential gut barrier dysfunction	Pilot scale, cross sectional, pediatric only, phenotype heterogeneity limits generalization	Fieten et al. (2018)
Integrative study (Animal + Human)	Reductions in Firmicutes and Bacteroidota, but enrichment in Actinobacteriota and Bifidobacterium	Gut dysbiosis, reduced diversity and altered metabolism.	Lack of adjustment for key clinical demographic and lifestyle confounders	Xu et al. (2025)
DNCB induced AD mouse model	Decreased Bifidobacterium and Ruminococcus	Skin barrier disruption, increased inflammation and altered gut microbial metabolism	Limited functional validation of microbial and metabolite roles in AD	Wang et al. (2024)
Murine oxazolone AD model	*Staphylococcus xylosus*	Microbiota alterations associated with mixed Th1/Th2 response similar to human AD dysbiosis	Shows microbiota involvement in AD-like inflammation but limited to induced mouse model conditions	Amar et al. (2022)

Collectively, these findings indicate that microbial colonization in early life influences immune development through compositional diversity and establishes a long-lasting immunological ‘setpoint’ through microbially derived biochemical signals. The balance between tolerogenic and pro-inflammatory immune responses during this critical period is mediated by microbial metabolites. These metabolites, including SCFAs, bile acids, and tryptophan derivatives, regulate immune cell differentiation, epithelial barrier integrity, and cytokine signaling pathways that are central to the pathogenesis of AD. Thus, the early life gut microbiome shapes immune programming not only through microbial presence but also through its metabolic output, providing a mechanistic link between microbial colonization and immune dysregulation.

### 5.1 Gut microbiota-derived immune modulation in AD

The gut microbiome plays a beneficial role in host physiology, including gut epithelial integrity, neurodevelopment, vitamin synthesis, and immune system maturation. This host immune system maturation occurs at both the mucosal and systemic levels through complex interactions with host cells and the metabolites they produce. Gut microbiota dysbiosis triggered by different factors, such as dietary shift, sex hormones, stress, host genetics, lifestyle behaviors, and prolonged antibiotic use, has been linked to several immune-mediated and metabolic disorders, including AD, inflammatory bowel disease, diabetes, obesity, and gastrointestinal conditions (Pessoa et al., 2023; Chen M. et al., 2024).

Numerous comparative studies have revealed that microbial imbalance alters immune signaling by modulating T helper (Th) and regulatory Treg cell responses and disrupting the balance between Th1, Th2, and Treg cells in patients with AD. This dysregulation often manifests as a Th2/Th1 imbalance in AD, with microbial dysbiosis leading to the overactivation of the Th1 and Th2 pathways and excessive proinflammatory cytokine production.

**Figure 2:**
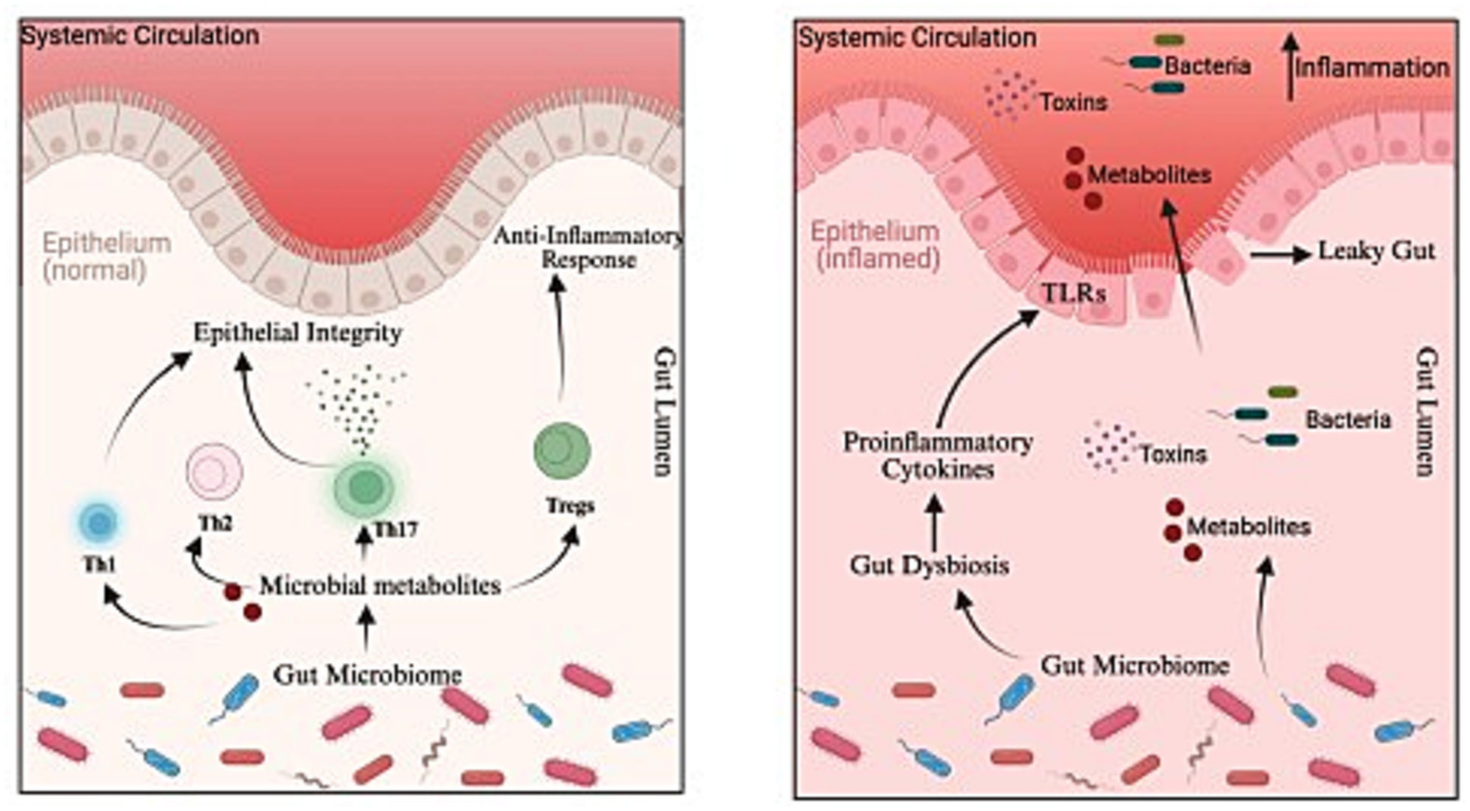
From immune tolerance to inflammation: The gut barrier’s role in health and disease: The left panel represents a healthy intestinal epithelium, indicating the role of the microbiome in the production of microbial metabolites that regulate immune cell subsets, support epithelial integrity, and maintain anti-inflammatory responses. The right panel shows gut dysbiosis in AD, characterized by altered microbial composition, impaired epithelial integrity (“leaky gut”) and translocation of toxins and metabolites.

The local gut microbiota plays a role in the maturation of gut-associated lymphoid tissue (GALT), enhancing mucus and antimicrobial peptide secretion to maintain barrier function. GALT comprises dendritic cells, T and B lymphocytes, plasma cells, and innate immune effectors, which are particularly concentrated in the colon mucosa or in Peyer’s patches. Microbial metabolites produced in the gut direct cell differentiation into various effector subsets, including Th1, Th2, Th17, and Tregs. For instance, segmented filamentous bacteria promote the Th1 and Th17 response, whereas *Bacteroides fragilis* and *Clostridia* promote the development of Tregs and IL-10 secretion, supporting the anti-inflammatory immune response (Fang et al., 2021; Salvadori and Rosso, 2024). Intestinal dendritic cells carry microbial antigens from the gut to the thymus and facilitate the generation of microbiota-specific T cells, which increases the ability of thymic T cells to distinguish commensal from pathogenic antigens. This reflects the pivotal role of the gut microbiota in the development of adaptive immune responses and the education of the immune system (Fang et al., 2021). Each type of helper T cell subset plays a role in shaping the immune response by producing cytokines that not only orchestrate effector functions but also suppress competing Th lineages. The gastrointestinal tract barrier function is supported and maintained in the gut by Th17-induced IL-17, IL-17F, and IL-22 produced by Th17 cells. These cytokines help clear pathogens from the mucosal surface and maintain epithelial integrity. Bacterial signals, including bacterial flagellin, unmethylated CpG DNA, and extracellular adenosine triphosphate, induce Th17 differentiation. IECs sense microbial signals via pathogen recognition receptors (PRRs) and relay them to ILCs. This crosstalk causes the secretion of IL-17 and IL-22, which are critical in maintaining barrier integrity but may also contribute to chronic inflammation under dysregulated conditions. In the context of AD, the key factor is the dominance of Th2/Th22 in the immune system. Th17 releases IL-17A, IL-17F, and IL-22, which stimulate keratinocytes to express S100A9 and S100A8; these factors play a role in inflammatory activities and amplify the Th2-mediated immune response. Therefore, the balance between Th1 and Th2 cells is important for immune homeostasis, and an imbalance can lead to chronic inflammation or allergic and autoimmune disorders. Certain bacterial strains, such as *Lactobacillus pentosus*, offer anti-inflammatory benefits, reducing the levels of proinflammatory cytokines (TNF-*α*, IL-17A, and IL-23), while *B. fragilis* and segmented filamentous bacteria stimulate the maturation of Tregs and Th17 cells, thereby enhancing immune stability and the host’s ability to fight infections. In contrast, gut dysbiosis activates proinflammatory cytokine production, alters the metabolic environment, and activates TLR-positive epithelial cells. This activation increases intestinal permeability by disrupting epithelial tight junctions and facilitating metabolite, toxins, and even bacterial translocation into the systemic circulation. These metabolites and microbes may sustain or promote proinflammatory cytokine production (Figure 2). Pathogenic taxa, such as *E. coli* and *C. difficile*, have been implicated in AD and eczema pathogenesis.

### 5.2 Microbial metabolites and immune system modulation

The gut microbiota produces a range of metabolites that significantly influence host physiology, particularly the immune system. Among the metabolites produced by tryptophan, SCFAs, trimethylamine (TMA), trimethylamine N-oxide oxide (TMAO) (Lee et al., 2018), and secondary bile acids have been identified as critical modulators of the immune response. Gut microbiota-derived metabolites have been implicated in the pathogenesis of both allergic and non-allergic diseases (Wu et al., 2022; Purevsuren et al., 2024). These metabolites improve allergic airway and skin inflammation by influencing immune cell function. Tryptophan regulates immunomodulatory effects across multiple tissues, including the lungs, gut, and skin (Yu et al., 2019; Purevsuren et al., 2024).

In neonates, a decrease in the variety of microbes in the intestinal microbiome and delayed colonization of Bacteroidetes have been associated with increased susceptibility to AD (Pessoa et al., 2023; Ozdemir and Oksuz, 2024). Collectively, gut microbial metabolites are essential for immune homeostasis (Zhang et al., 2023), inflammation regulation, and gut health. Gut microbe metabolites not only modulate immune cell behavior but also strengthen the intestinal barrier function, adapt the host’s response to environmental stimuli, and contribute to overall well-being. Therefore, the equilibrium of the gut microbiota is critical; disruptions of this balance lead to intestinal permeability and compromise the integrity of the intestinal barrier. Disruption of the intestinal barrier allows metabolites to enter the bloodstream and reach the skin and provokes a strong Th2 response, which worsens the immune system dysfunction and disease onset. Further mechanistic studies are required to elucidate the complex interactions between gut microbes, their metabolites, and the host immune system (Chen M. et al., 2024; Shen et al., 2024). Under normal physiological conditions, the gut microbiome maintains the integrity of the gut epithelium by preventing luminal cytokine translocation. However, dysbiosis induced by pathogens, such as *E. coli* and *C. difficile*, can disrupt this barrier function, leading to cytokine leakage, microbial proteolysis, and prolonged colonic transit time. The resultant increase in protein fermentation results in the production and accumulation of trimethylamine, branched-chain fatty acids (e.g., 2-methylbutirate, isobutyrate, and isovalerate), organic acids, gases, and trace amounts of phenols, indoles, and ammonia, which collectively cause an increase in the luminal pH and contribute to intestinal inflammation. Such a dysbiotic shift causes the leakage of pathogen-associated molecular patterns, including lipopolysaccharides, ultimately triggering systemic low-grade inflammation. These disruptions are closely associated with eczema and asthma (Salvadori and Rosso, 2024; Figure 2). Trimethylamine N-oxide (TMAO) increases the levels of proinflammatory cytokine namely IL-1β, IL-6, and IL-18, through the activation of the NLRP3 inflammasome, and plays a role in a variety of inflammatory diseases, such as psoriasis. Currently, there is a lack of research on how TMAO plays a role in AD, and investigations are needed to fully elucidate the involvement of this bacterial metabolite in AD pathogenesis (Pessoa et al., 2023).

Gut microbiota-derived metabolites influence the activation and differentiation of B and T cells and promote the protective antibody immune response. Differentiation of Naïve T cells into Th1, Th2, Th17, and Foxp3 + T cells are strongly dependent on gut microbial cues. Tregs control inflammation and maintain immune tolerance by controlling the cellular activities of basophils, eosinophils, and mast cells and by inducing IgG production and IgE suppression, respectively (Jung Eun and Hei Sung, 2019).

Several gut resident genera, such as *Bifidobacterium*, *Clostridium*, *Bacteroides*, *Streptococcus,* and *Lactobacillus*, produce SCFAs, such as butyric acid and propionic acid, which are potent Treg inducers. Thymus-derived Tregs (tTregs) develop centrally, whereas pTregs differentiate from naïve T cells in peripheral tissues and suppress mucosal Th2-mediated inflammation. *B. fragilis promotes* pTreg development by producing the symbiosis factor polysaccharide A (Jung Eun and Hei Sung, 2019). Modulation of the gut microbiome to favor the expression of Th1 cytokines enhances the integrity of the gut mucosal barrier by the production of polyamines and SCFAs, such as butyrate. This microbial-driven immune response rebalancing offers therapeutic potential for alleviating AD symptoms (Chen M. et al., 2024).

### 5.3 Microbial metabolites in gut–skin axis signaling

The gut microbiota produces a wide range of metabolites, particularly three key classes of microbiota-derived metabolites, SCFAs, tryptophan metabolites, and secondary bile acid, which are important in the context of immune regulation within the gut–skin axis. These metabolites play important roles in shaping immune cell differentiation, maintaining epithelial barrier function, and regulating inflammatory signaling pathways. The following sections explore each metabolite class in detail, emphasizing their roles in immune modulation and AD pathogenesis.

#### 5.3.1 Role of SCFA in immune system modulation

SCFAs, the primary metabolites produced through bacterial fermentation of dietary fibers in the gut, are the most well-studied microbial products due to their role in immune regulation and host metabolism (Rooks and Garrett, 2016; Purevsuren et al., 2024). SCFAs modulate local and systemic immune responses. SCFAs regulate immune cell behavior by epigenetic modification of cellular functions and contribute to the prenatal programming of allergic diseases (Yip et al., 2021; Pessa-Morikawa et al., 2022). They also serve as the main energy substrate for enterocytes and colonocytes, reduce luminal pH, stimulate and mature Tregs, and have anti-inflammatory properties (Salvadori and Rosso, 2024; Zhou et al., 2024). SCFAs, mainly butyrate, propionate, acetate, isobutyrate, valerate, and isovalerate, produced by fermentation of dietary fiber by gut bacteria, contain anti-inflammatory properties (Lee et al., 2018; Xiao et al., 2023). *Eubacterium*, *Bifidobacterium, Coprococcus*, *Cutibacterium (Propionibacterium), and Blautia* produce SCFAs. Table 2 lists the different types of SCFAs produced by different bacterial genera.

**Table 2:** Short-chain fatty acids and their associated bacterial producers.

SCFAs	SCFAs producing bacterial genera
Acetate	*Akkermansia*, Firmicutes, and Bacteroidetes (Pessoa et al., 2023).
	*Akkermansia muciniphila, Blautia hydrogenotrophica,* and *Bifidobacterium longum* (Purevsuren et al., 2024).
Propionate	Bacteroidota (Pessoa et al., 2023; Ozdemir and Oksuz, 2024).
	*Roseburia*, *Bifidobacterium*, and *Bacteroides* (Jung Eun and Hei Sung, 2019; Xu et al., 2020; Purevsuren et al., 2024) Firmicutes (Jung Eun and Hei Sung, 2019)
Butyrate	*Faecalibacterium*, *Eubacterium*, *Anaerobutyricum* (Pessoa et al., 2023; Ozdemir and Oksuz, 2024; Purevsuren et al., 2024)

The gut microbiome is enriched in pathways associated with SCFAs metabolism. Individuals with AD have decreased populations of SCFA-producing bacteria. The decreased production of SCFAs may cause intestinal barrier breakdown and compromise, leading to increased intestinal permeability and inflammation, which triggers AD. Oral administration of SCFAs alleviates AD progression due to their anti-allergic and anti-inflammatory properties. It can restore the balance between intestinal T-cell subpopulations (Th17/Treg) (Xiao et al., 2023).

In patients with AD, especially children, a low level of fecal SCFA indicates a reduced abundance of SCFA-producing bacteria. For instance, infants aged 6–24 months with AD show lower butyrate and valerate levels than healthy infants (Park et al., 2020). Similarly, children under 3 years of age suffering from AD exhibited lower amounts of propionate and butyrate. AD-like symptoms induced by transdermal injection of 2,4-dinitrochlorobenzene correlated with reduced SCFA levels in mice compared with healthy littermates. The AD symptoms were relieved by SCFA normalization in these mice models (Chun et al., 2021; Xiao et al., 2023). Decreased diversity and loss of SCFA-producing bacteria are associated with epithelial damage and disease severity (Nylund et al., 2015).

In AD skin, *S. aureus* promotes inflammation by binding and activating TLR-2, resulting in IL-4-mediated inhibition of IL-10. SCFAs play a role in the improvement of skin microflora and also restrict the migration of immune cells and the production of TNF-*α*, and IL-6 (Xiao et al., 2023). Birth cohort studies have demonstrated that children with higher SCFA levels during infancy or at younger ages are less likely to develop allergic sensitization and AD (Purevsuren et al., 2024).

SCFAs play a context-dependent immunomodulatory role. Under normal conditions, SCFAs maintain the immune system, whereas during infections, they can increase the effectiveness of the immune system to fight harmful invaders. In immune and epithelial cells, SCFAs inhibit HDACs and block NF-kB signaling/activation, thereby reducing the expression of pro-inflammatory cytokines and chemokines (CCL2 and CCL5) within 24 h (Rooks and Garrett, 2016; Purevsuren et al., 2024). SCFAs mediated HDAC inhibition and Gpr41 interaction by modulating the aryl hydrocarbon receptor (AhR) and hypoxia-inducible factor-1α (HIF-1α), activating CD4^+^ T cells and ILCs, and promoting IL-22 production, a cytokine from the IL-10 family involved in the anti-inflammatory response and tissue repair. It also affects dendritic cell function, Treg differentiation, and airway epithelial integrity to improve barrier integrity (Hu et al., 2022; Trompette et al., 2022; Purevsuren et al., 2024; Zhou et al., 2024).

SCFAs bind with G protein-coupled receptors (GPR41, GPR43, and GPR109a), which are expressed on immune and enteroendocrine cells. This activates the intracellular signaling cascade (Rooks and Garrett, 2016; Purevsuren et al., 2024) to stimulate glucagon-like peptide 1. This leads to increased energy expenditure, improved glucose metabolism, increased insulin secretion, and reduced food intake (Salvadori and Rosso, 2024). In intestinal macrophages, SCFAs induce oxygen-based pathway (OXPHOS) oxidative phosphorylation over glycolysis to promote energy production and enhance antimicrobial capabilities (Rooks and Garrett, 2016; Xiao et al., 2023; Purevsuren et al., 2024). SCFAs maintain the balance of CD4^+^IL17^+^ T cells/CD4^+^FOXP3^+^ Treg cells and affect ILC3s in the intestinal mucosa and IL-17 and IL-22 expression by ILC3s. SCFAs, including butyrate, propionate, and acetate, interact with the intestinal epithelial barrier to mediate anti-inflammatory and immunomodulatory effects. In the colon, butyrate and propionate promote DC activation, T cell G protein signaling, and Treg accumulation and reduce inflammation. They achieve this by inhibiting immune cell migration, reducing ROS, and attenuating inflammatory signals. In animal models, high-fiber diets increase SCFA levels and inhibit Th2 cell differentiation and allergic inflammation. Linoleic acid and 10-hydroxycis-12-octadecenoic acid modulated the gut microbiome and alleviated AD symptoms in mouse models In conclusion, SCFAs, the key metabolites of microbial fermentation, are key regulators of immune response homeostasis. SCFAs prevent inflammation, maintain intestinal barrier function, and modulate both local and systemic immune responses (Chen M. et al., 2024; Zhou et al., 2024; Pessoa et al., 2023; Xiao et al., 2023; Purevsuren et al., 2024).

**Figure 3:**
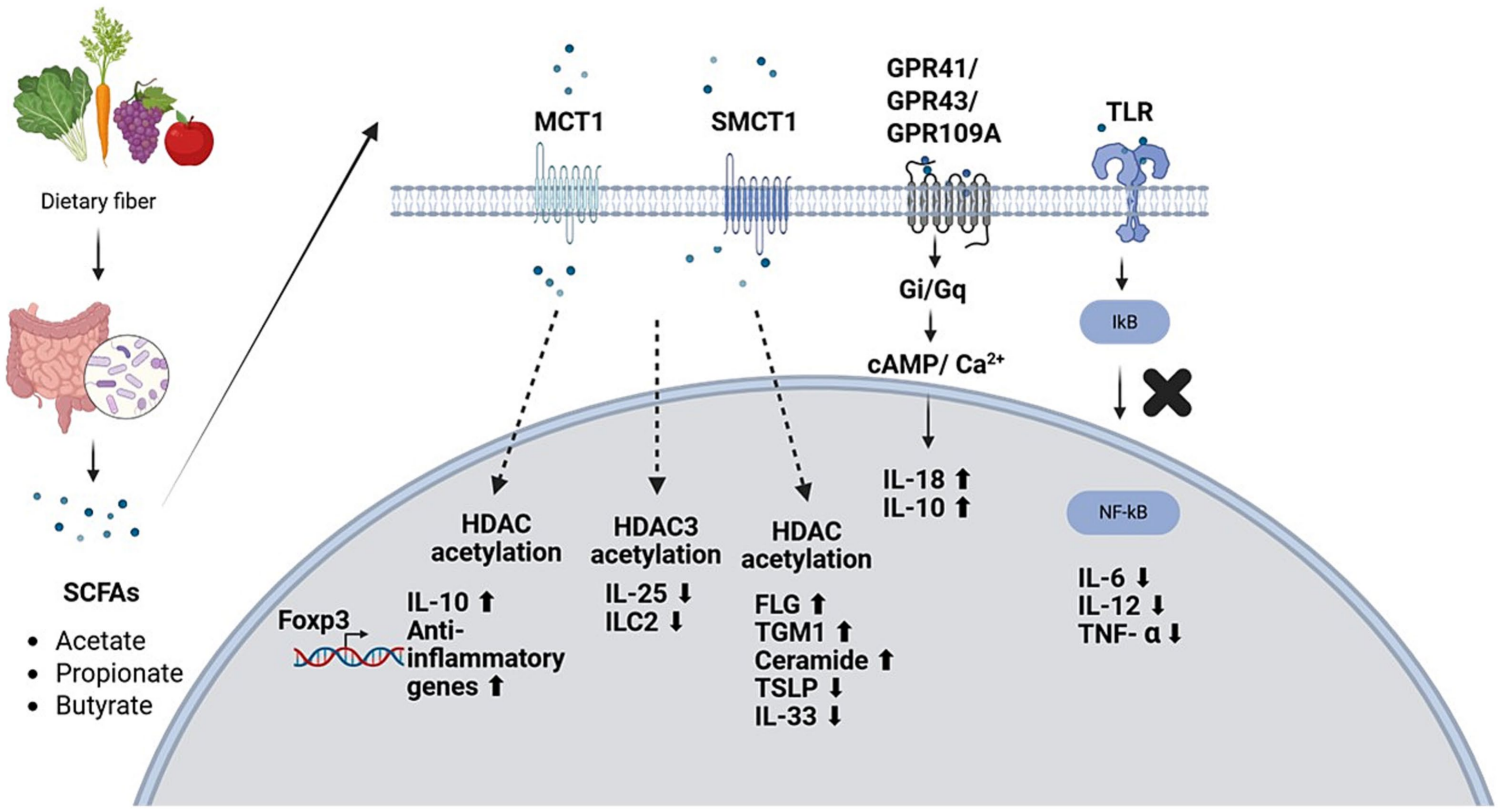
Mechanisms by which short-chain fatty acids control type-2 immunity and skin barrier function along the gut-skin axis. Short-chain fatty acids (SCFAs) produced by the colon microbiota enter the circulation and go to the peripheral tissues, such as the skin. Butyrate has been shown to modulate intestinal epithelial progenitors by suppressing HDAC3, restraining tuft cell differentiation, and inhibiting IL-25 synthesis, thereby reducing ILC2 proliferation and subsequent type-2 cytokine expansion. Simultaneously, SCFAs signal through GPCRs (FFAR2, FFAR3, and GPR109A) to regulate dendritic cell development and Th2 effector response inhibition. Butyrate improves mitochondrial metabolism and suppresses HDAC in keratinocytes, resulting in elevated expression of barrier proteins, including filaggrin and transglutaminase-1, and induces ceramide production. In addition, SCFAs directly inhibit the inflammatory stimulation of cutaneous immune cells. Collectively, these mechanisms with epigenetic and receptor-mediated actions inhibit type-2 inflammation, enhance epithelial barrier integrity, and protect against AD.

Short-chain fatty acids act as signaling molecules through two tightly interconnected mechanisms: inhibition of histone deacetylases (HDACs) and activation of dedicated G-protein-coupled receptors (GPCRs) (Figure 3).

##### 5.3.1.1 Epigenetic reprogramming via HDAC inhibition

Histone acetylation on the lysine residues of histones H3 and H4 converts chromatin from a repressive to a transcriptionally permissive state. Histone acetyltransferases and HDACs maintain this balance. Among SCFAs, butyrate is the strongest endogenous HDAC inhibitor of the SCFAs, and it acts physiologically at concentrations attained by the colon (Koh et al., 2016). The epigenetic modification of HDAC directly influences the Foxp3 locus that produces the transcription factor that defines the lineage of regulatory T cells (Tregs). HDAC inhibition mediated by SCFA increases acetylation of Foxp3 regulatory regions and *de novo* Foxp3 expression in naive CD4 + T cells under TGF-B signaling conditions. Notably, it is the conserved non-coding sequence 1 (CNS1) enhancer element of the Foxp3 gene, which is necessary to differentiate peripheral (extrathymic) Tregs, but not thymic Tregs, and SCFAs selectively reinforce peripheral tolerance (Arpaia et al., 2013). The sensitivity of cells to SCFA-mediated HDAC inhibition is determined by the metabolic state (Furusawa et al., 2013). Effector T cells (Th1, Th2, and Th17) are dependent on aerobic glycolysis, a metabolic organization that increases the intracellular levels of SCFAs and improves their HDAC-inhibitory properties (Shi et al., 2011). Conversely, oxidative phosphorylating cells can use butyrate as an energy source that restricts nuclear buildup. This context-dependent action of butyrate can be explained by metabolic gating and highlights the ability of SCFAs to selectively induce Treg differentiation and limit the overgrowth of effector responses. Butyrate inhibits proinflammatory mediators, such as TNF-*α* and IL-6, in lamina propria macrophages, in part by inhibiting the NF-κB pathway (Ottria et al., 2026). SCFAs suppress the differentiation of bone-marrow precursors in dendritic cells (DCs), suppressing the production of IL-6 and IL-12 in favor of a tolerogenic phenotype, which induces Foxp3 + Tregs instead of effector T-cell priming. Such integrated actions create a regulatory immunologic milieu of augmented IL-10-producing T cells and suppressed production of inflammatory cytokines that oppose the dysregulated Th2-biased immunity found in AD (Koh et al., 2016).

##### 5.3.1.2 GPCR-mediated signaling

Simultaneously with the action of intracellular HDAC inhibition, SCFAs act via membrane-bound g protein receptors (GPCRs). FFAR2 (GPR43) is a dual Gi/o- and Gq-coupled receptor that is activated by acetate and propionate at micromolar levels (Brown et al., 2003). It is found in colonic epithelial cells, neutrophils, and a variety of leukocyte subsets where it controls chemotaxis, cytokine production, and epithelial hormone secretion. FFAR3 (GPR41) only interacts with Gi proteins and is most sensitive to propionate and butyrate (Le Poul et al., 2003). SCFAs also regulate cAMP intracellular levels and kinase signaling cascades via these receptors, which affect immune cell recruitment and differentiation. GPR109A (also known as HCA2) is a high-affinity butyrate receptor located on the apical epithelial cell surface of the intestine, macrophages, and DCs (Thangaraju et al., 2009). GPR109A activation stimulates IL-18 secretion in epithelial cells and facilitates IL-10 secretion and Treg differentiation, which are important for intestinal homeostasis (Singh et al., 2014). NLRP3 inflammasome activation has also been associated with GPR43 and GPR109A activation induced by a high-fiber diet to further combine microbial metabolism with innate immune regulation (Macia et al., 2015). These receptor-mediated mechanisms work in coordination with epigenetic processes to ensure that commensal tolerance is maintained while preserving the capacity to respond to pathogens (Koh et al., 2016).

##### 5.3.1.3 Effects on the skin barrier and cutaneous immunity

SCFAs are partly absorbed in the gut and enter the bloodstream to reach distant organs and tissues, such as the skin, and act directly on keratinocytes to reduce the expression of the pro-inflammatory cytokine IL-6 and the intercellular adhesion molecule-1 (ICAM-1) leading to decreased recruitment of monocytes to the epidermis to alleviate AD (Xiao et al., 2023). Moreover, SCFAs can boost the Th1 and Th17 cell population during active immune responses (Pessoa et al., 2023).

Butyrate in keratinocytes is converted to acetyl-CoA, which increases the mitochondrion’s oxidative metabolism and stimulates cellular differentiation and barrier formation. HDAC inhibition in keratinocytes increases the structural protein expression of filaggrin (FLG), transglutaminase-1 (TGM1), and ceramide synthesis, which enhances the stratum corneum and decreases transepidermal water loss (Blicharz et al., 2025). Improved barrier integrity reduces the penetration of allergens and release of alarmins by epithelial cells (TSLP, IL-25, IL-33) that play pivotal roles in Th2 activation in AD. The cutaneous immune cells also react directly to SCFAs. Macrophages, T lymphocytes, and innate lymphoid cells within the skin express SCFA-responsive receptors and exhibit attenuated pro-inflammatory activation following SCFA exposure. In addition, SCFAs reduce inflammatory reactions triggered by resident skin bacteria, which supports immune homeostasis at the epidermal barrier (Kim and Baker, 2026).

##### 5.3.1.4 The specific role of butyrate in immune regulation and AD

AD severity is negatively correlated with the abundance of butyrate-producing bacteria, such as *Bifidobacterium*, *Coprococcus*, *Blautia*, *Eubacterium*, and *Propionibacterium* (Park et al., 2020; Xiao et al., 2023; Reddel et al., 2019; Lee M.-J. et al., 2022). The gut bacterial species *F. prausnitzii*, a known butyrate producer, is beneficial for gut health and has been observed in *in vivo* studies to improve AD markers, such as dermatitis score and scratching behavior, and reduce IgE levels (Lee Y. et al., 2022). In AD, intestinal barrier impairment and increased permeability are common. SCFAs produce gram-positive anaerobes that ferment carbohydrates into butyrate, which upregulates the expression of tight junction proteins by inhibiting proinflammatory signaling pathways such as nuclear factor kappa B (NF-kB) and, as a result, enhances intestinal barrier integrity. In the colon, propionate and butyrate promote the proliferation and activation of Tregs by activating the dendritic cell (DCs) and G protein signaling pathways, which ultimately increases the ability of Tregs to suppress the proliferation of effector CD4 + T cells (Nylund et al., 2015). Tregs are a specialized subset of T cells that can inhibit the activation of effector T cells. Tregs have immunosuppressive properties and maintain immune tolerance and homeostasis (Zhu et al., 2019). Butyrate enhances anti-inflammatory factor expression by inhibiting GPr41 and HDAC to promote IL-22 production (Chen M. et al., 2024; Zhou et al., 2024). In the gut, butyrate activates the peroxisome proliferator-activated receptor gamma, promoting *β*- oxidation, oxygen consumption, thereby maintaining an anaerobic environment in the gut lumen (Salvadori and Rosso, 2024).

#### 5.3.2 Tryptophan derivatives and AD

Digesting proteins in the intestine yields amino acids, including tryptophan (Trp), an essential amino acid involved in multiple host metabolic and immunological processes (Fang et al., 2022). Notably, patients with AD have reduced Trp metabolite levels in their skin (Purevsuren et al., 2024). Several commensal gut-residing bacteria, such as *Bifidobacterium*, *Clostridium*, *Bacteroides*, and *Lactobacillus, metabolize* tryptophan, primarily through the indole pathway, and ultimately affect host health (Xu et al., 2022). The resulting metabolites include indole and its derivatives, such as indole acetic acid (IAA), indole-3-acrylic acid (IA), indole-3-lactic acid (ILA), indole-3-propionic acid (IPA), and indole-3-carbaldehyde (I3C). These compounds function as natural ligands for the AHR, a transcription factor involved in immune regulation and epithelial homeostasis (Sun et al., 2020; Fang et al., 2022). Tryptophan-derived metabolites, particularly indole-3-aldehyde (IAld), exert immunomodulatory and anti-inflammatory effects in the central nervous system and mucosal environments (Chen M. et al., 2024). Trp metabolite derived by the skin microbiome and indole-3-aldehyde (IAlD) activates AhR in keratinocytes, which leads to the suppression of TSLP production and enhancement of the epidermal barrier in AD (Yu et al., 2019; Fang et al., 2022).

The compounds produced in the indole pathway play a role in AhR activation to modulate host immunity by enhancing glucagon-like peptide-1 (GLP-1) production and suppressing pro-inflammatory pathways (Xu et al., 2022). AHR signaling is important for immune modulation at barrier sites, such as the skin and gut, where it interacts with key immune cells, like dendritic cells and innate lymphoid cells, to fine-tune the immune response (Fang et al., 2022).

Gut commensal bacteria play a role in systemic immune regulation by tryptophan metabolism. For instance, *a gut bacterium, Lactobacillus reuteri*, synthesizes I3A, which activates the AhR receptor and triggers IL-22 production. IL22 is pivotal in maintaining mucosal barrier integrity and preventing aberrant immune system activation (Zelante et al., 2013). Similarly, *B. longum* upregulates tryptophan metabolism and increases I3A levels in feces and serum, resulting in reduced AD symptoms (Fang et al., 2022). Collectively, these findings indicate the protective role of microbial-derived metabolite especially IAlD and I3A, in the modulation of epithelial barrier function and immune homeostasis in AD.

#### 5.3.3 Aryl hydrocarbon receptor signaling pathway

**Figure 4:**
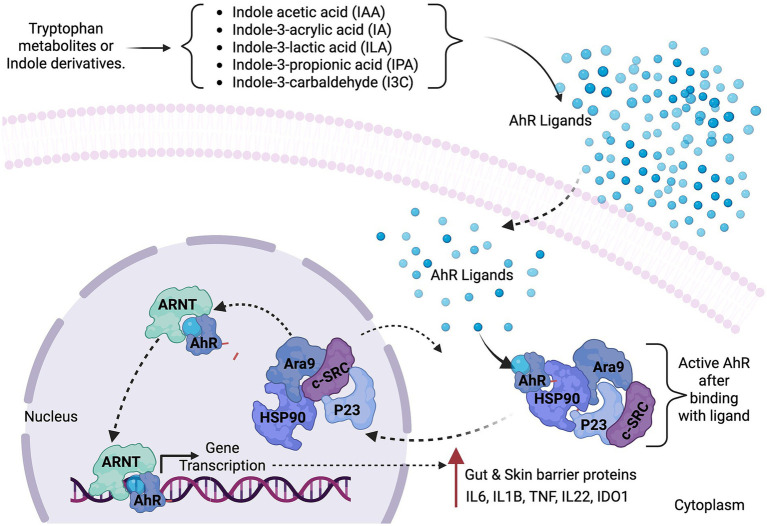
Tryptophan-derived metabolites control immune activities through the aryl hydrocarbon receptor (AhR). Microbial tryptophan metabolites are AhR ligands. In the inactive state, AhR binds to Hsp90 and co-chaperone p23 in the cytoplasm. Ligand binding initiates AhR release, leading to the nuclear translocation and transcriptional activation of target genes that facilitate regulatory T-cell differentiation and production of barrier-protecting cytokines, such as IL-22, and immune homeostasis maintenance.

The AHR is a transcription factor that regulates various physiological and pathological processes, including detoxification, metabolism, cell proliferation, differentiation, inflammation, and immune responses (Figure 4). AHR can activate both canonical and noncanonical signaling pathways. In the canonical signaling pathway, AHR agonists exert their therapeutic potential in AD. In the cytoplasm without the ligand, AHR is present as part of a protein complex. The complex consists of the AhR-interacting protein Ara9, c-SRC protein kinase (c-SRC), heat shock protein HSP90, and p23. Conformational changes occur in AHR upon binding with a ligand, exposing AHR to protein kinase C-mediated phosphorylation or its nuclear translocation site, which promotes its translocation toward the nucleus. In the nucleus, AHR dissociates from the protein complex and interacts with the AhR nuclear translocator (ARNT) to form the AhR-ARNT heterodimeric complex. This complex then binds with a specific DNA sequence (5′-TNGCGTG-3′), known as dioxin-responsive elements (DREs) or xenobiotic-responsive elements (XREs), to regulate gene transcription. The AHR–ARNT complex also induces the expression of CYP1A1, CYP1A2, CYP1B1, NAD(P)H-quinone oxidoreductase, and AHRR. The AHRR protein acts as a negative feedback regulator in the AHR signaling pathway to modulate AHR-dependent gene transcription. The CYP1A1 protein assists in the biotransformation and degradation of AHR ligands (Napolitano et al., 2021; Wazir and O’Toole, 2025; Riaz et al., 2022). In the noncanonical pathway, nuclear AHR binds without ARNT and leads to the expression of genes responsible for maintaining homeostasis. Some AHR-regulated genes contain nonconsensus XRE sequences but a repeated tetranucleotide motif, which eliminates the need for interaction with ARNT (Napolitano et al., 2021).

#### 5.3.4 Bile acids

Primary bile acids are synthesized in the liver and subsequently conjugated with taurine or glycine before being transported to the duodenum. A portion of these conjugated bile acids escapes enterohepatic reabsorption and reaches the colon, where the gut microbiota enzymatically converts them into secondary bile acids such as lithocholic acid (LCA), deoxycholic acid (DCA), and ursodeoxycholic acid (UDCA). The key species associated with these conversions are *C. hylemonae*, *E. lenta* (*Eubacterium lentum*), *C. scindens*, *R. gnavus*, and *B. fragilis* (Purevsuren et al., 2024).

Mechanistically, secondary BAs may regulate immunity by two mechanisms: (i) receptor-mediated signaling by binding BARRs, including the Farnesoid X receptor (FXR) and the G protein-coupled BA receptor 1 (GPBAR-1, also referred to as TGR-5) (Kawamata et al., 2003), which leads to the activation or inhibition of various immune-modulatory programs. (ii) Direct modulation of cellular transcriptional programs through ligand-dependent interactions with nuclear receptors such as RORγt (Hang et al., 2019). Bile acids exert their immunomodulatory effect by interacting with specific host receptors, such as FXR and GPBAR1/TGR5 (Purevsuren et al., 2024). FXR has a strong anti-inflammatory and protective barrier effect. Its stimulation inhibits pro-inflammatory signaling by multilayered pathways, which encompass the direct sequestration and induction of proteasomal degradation of the NF-κB p65 subunit and the cytokine transcription of TNF-*α*, IL- 6 and IL- 1β by chromatin-bound SHP complexes (Ha et al., 2023). GPBAR1 binds a ligand and associates with the Gαs protein, leading to adenylyl cyclase activation and a resultant increase in intracellular cAMP (Zangerolamo et al., 2025). The key to GPBAR1-mediated immunosuppression is this second messenger. Mechanistically, increased cAMP stimulates protein kinase A phosphorylation of the cAMP-response element-binding protein. More importantly, protein kinase A activation disrupts the NF-κB signaling pathway. In the absence of GPBAR1 activation, inflammatory stimuli cause the phosphorylation and degradation of IκBα, allowing the p65/p50 NF-κB complex to translocate into the nucleus and induce the expression of proinflammatory genes. GPBAR1 activation inhibits this translocation. This inhibition prevents the expression of key cytokines such as TNF-α, IL-1β, IL-6, and MCP-1 (Pols et al., 2011). The GPBAR1-initiated cAMP-protein kinase A signaling cascade results in the phosphorylation of NLRP3 at particular serine residues, which ubiquitylates NLRP3 and directs it to proteasomal degradation or inhibits its assembly (Guo et al., 2016). In human macrophages, the activation of GPBAR1 by taurolithocholic acid (TLCA) causes the downregulation of expression of pro-inflammatory signaling and facilitates a shift from the classically activated pro-inflammatory macrophage M1 to the regulatory macrophage M2b phenotype. Similarly, the activation of the nuclear FXR receptor by ursodeoxycholic acid induces an immunosuppressive phenotype in dendritic cells and contributes to the modulation of Treg cell populations.

**Figure 5:**
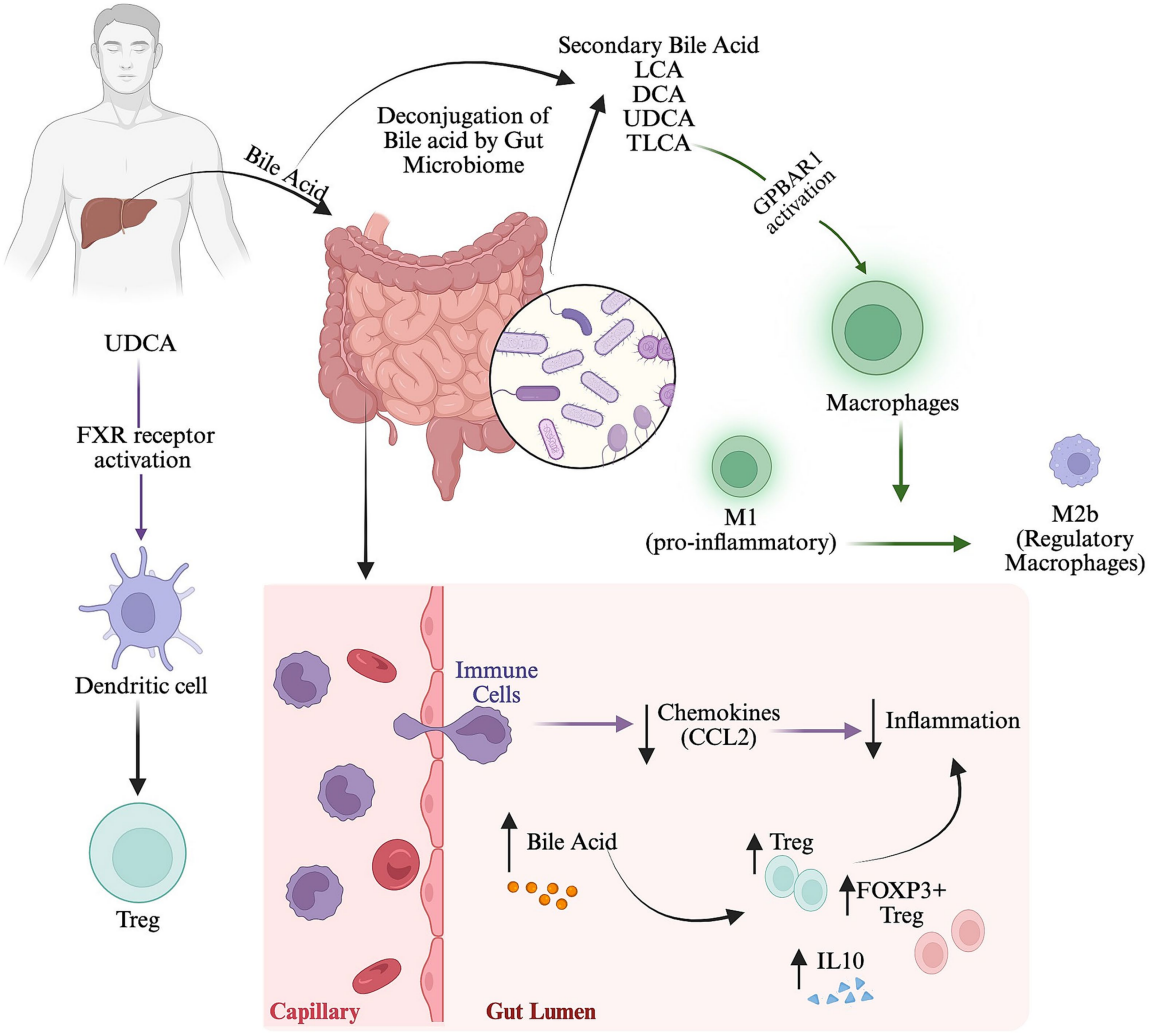
Secondary bile acid-mediated immune regulation in the gut. Schematic overview of gut microbiota-mediated bile acid metabolism and its potential role in regulating intestinal immune responses.

*Bifidobacterium* and *Lactobacillus* species control inflammation by producing microbial bile salt hydrolases, which catalyze the conversion of bile acids to secondary bile acids and act as NOD-like receptor thermal protein domain associated protein 3 (NLRP3) inflammasome inhibitors, thus controlling inflammation. Elevated levels of bile acid in the gut are associated with increased recruitment of innate immune cells from the blood to the intestine and reduced expression of the chemokine CCL2, which is implicated in the pathogenesis of AD. Furthermore, a higher bile acid level was correlated with high Treg, IL10, and FOXP3 + Treg cell generation (Figure 5). In humans, BAs also act as signaling molecules and interact with farnesol X receptor (FXR), Takeda G protein receptor 5 (TGR5), and the sphingosine-1-phosphate receptor 2 (S1PR2).

TGR5 activation suppresses pro-inflammatory cytokine production and reduces macrophage infiltration. On the other hand, S1PR2 activation triggers mast cell degranulation, which increases the production of inflammatory cytokines and S1P. A higher level of circulating S1P in the blood promotes the Th2 immune response, causing more Th2 and Th17 cells to develop and increasing the M2/M1 macrophage ratio. Thus, bile acids may exert either anti-inflammatory or proinflammatory effects depending on the receptors they interact with and affect immune cell activity and inflammatory responses. In the gut, B cells also contribute to bile acid homeostasis. Impaired humoral immunity can disrupt this regulatory axis, thereby predisposing individuals to intestinal inflammatory diseases. B cells and bile acid significantly influence the severity and progression of intestinal inflammatory disease (Purevsuren et al., 2024).

Bile acids and their metabolites enter the systemic circulation and act on skin-resident immune cells, including keratinocytes, DCs, and T cells, through receptors such as FXR and TGR5. Altered bile acid profiles modulate cytokine production, immune cell infiltration, and epidermal barrier integrity in inflammatory skin diseases, including AD and psoriasis. In AD, bile acid signaling influences pruritus, immune polarization, and barrier dysfunction. For example, TGR5 activation regulates itch-related ion channels such as TRPA1 and TRPV1, whereas S1PR2 signaling promotes inflammatory cytokine production and amplifies Th2/Th17-mediated inflammation. Dysregulated bile acid metabolism is associated with impaired skin barrier function, increased keratinocyte dysfunction, and enhanced inflammatory responses, all of which are central to the pathogenesis of AD (Ma et al., 2025).

## 6 Emerging therapeutic approaches in AD

The therapeutic benefits of probiotics, prebiotics, synbiotics and microbial derived metabolites have attracted growing interest in treatment of AD. Nevertheless, the potential and the mechanistic insights, the clinical evidence is not yet conclusive and heterogeneous and cannot be used to make definite conclusions about the efficacy.

### 6.1 Prebiotics

Prebiotics are non-digestible food that selectively stimulate beneficial gut microorganisms and improve host health (Gibson and Roberfroid, 1995). In 2010, a study revealed that the prevalence of AD could be reduced in infants with low atopic risk when specific prebiotic combinations were added to infant formula within the first year of life. It was a mix of long-chain fructo-oligosaccharides (lcFOS), pectin-derived acidic oligosaccharides (pAOS), and short-chain oligosaccharides (scGOS). This implies that prebiotics could be preventive in infants at low risk for AD (Grüber et al., 2010). Slight improvements in disease severity in terms of decreased SCORAD scores and positive correlation between enhanced *F. prausnitzii* abundance and clinical improvement in young children were shown using small randomized controlled trials with kestose. These effects however were not recorded uniformly across all age groups and are limited by small sample sizes, study period and absence of mechanistic evidence. Thus, existing evidence indicates potential benefit, but is inadequate to draw solid clinical conclusions (Koga et al., 2016).

### 6.2 Probiotics

Probiotics, which are live microorganisms that provide health benefits to the host, have been widely studied in the treatment and prevention of AD. Clinical studies on the role of probiotics in AD have had mixed results conducted in a plethora of studies. Cochrane review of 39 randomized controlled trials found little or no difference in eczema symptoms and indicated limited therapeutic benefit (Pan and Liu, 2026). Conversely, some meta-analyses have indicated a decrease in AD incidence with probiotic supplementation, especially when used during pregnancy and early infancy, which indicates a preventive effect. Mixed-strain probiotics can reduce the SCORAD scores, but with inconsistent results based on strain composition and patient age. For example, some strains, like *Lactobacillus paracasei* and *Lactobacillus sakei*, have demonstrated clinical responses but *Lactobacillus rhamnosus* has elicited mixed responses (Tan-Lim et al., 2021).

Recent meta-analyses also provide further support concerning a decrease of AD risk among high-risk children younger than six years, with some studies providing different results in terms of clinical outcomes including SCORAD, quality of life and cytokine levels, e.g., IgE, TNF-*α* and IL-4 (Umborowati et al., 2022). These case studies, in general, indicate that probiotics can be more effective as a preventive than as a therapeutic agent, and the effects depend on the specificity of the strain, time of administration and characteristics of the population.

### 6.3 Synbiotics

Synbiotics, composites of prebiotics and probiotics have been considered an option in the treatment of AD, with several clinical trials reporting a positive effect. Indicatively, children trials have demonstrated that symbiotic enriched formulas containing *Bifidobacterium* M-16 V and GOS/FOS can decrease the severity of disease, as reflected by a lower SCORAD score in infants with IgE-related AD (van der Aa et al., 2011). Other studies have shown that the composition of gut microbiota is positively changed in infants with suspected food allergies after synbiotic supplementation (Candy et al., 2018). Besides, breast milk, which is regarded as a natural synbiotic, could also help to decrease the risk of allergy because of its probiotic and prebiotic contents (Zubeldia-Varela et al., 2022).

Overall, these case studies indicate that synbiotics could have synergistic effects on the regulation of gut microbiota and better AD-related outcomes, especially in infants and high-risk groups. Nevertheless, the results are inconsistent, and the existing evidence has small samples and heterogeneity of studies. So, although synbiotics show promise, additional large-scale, and well-designed randomized controlled studies are required to demonstrate their clinical effectiveness and the underlying mechanisms (Pan and Liu, 2026).

### 6.4 Microbial metabolites

The activity of immune cells is affected by microbial metabolites like SCFAs and tryptophan-derived indoles. They assist in keratinocyte differentiation to keep the epithelial integrity intact (Trompette et al., 2022). This process can be regulated by dietary factors with tryptophan-rich diets promoting the generation of AhR-stimulating metabolites that enhance immune homeostasis and barrier maintenance. On the other hand, low amounts of these metabolites are linked to the loss of barrier integrity and inflammation, and thus, diet-induced changes in gut-derived metabolites can potentially have preventive and curative effects in AD (Kim et al., 2024).

Glycomacropeptides (GMPs) are bioactive peptides derived from dietary proteins that are beneficial to human health. Glycopeptides prophylactic feeding causes production of SCFA, which prevents and reverses AD-like skin lesions in rats. Studies show that the protective effect of GMPs on the skin barrier may be mediated by the direct effects of acetate and butyrate on local skin cells. Collectively, SCFAs may reduce the symptoms of inflammatory skin diseases (Xiao et al., 2023).

## 7 Limitations

The therapeutic translation of gut microbiota-derived metabolites, such as SCFAs, with strong immunomodulatory effects in preclinical models remains limited because of the challenges in dose standardization, delivery, and bioavailability. Not all studies report positive outcomes, and some fail to demonstrate significant clinical improvements. These inconsistencies underscore the complexity of host gut microbiome interactions and emphasize the need for well-designed, large-scale, and standardized clinical trials before these approaches can be reliably integrated into clinical practice.

## 8 Conclusion

The interplay between gut microbiota, microbial metabolites, and host immune responses offers novel insights into the pathogenesis of AD. The gut-skin axis plays a critical role in AD, as alterations in the gut microbiota composition and reduced abundance of microbial metabolites, such as SCFAs, tryptophan derivatives, and secondary bile acids, contribute to systemic immune dysregulation and epithelial barrier dysfunction. These microbe-derived metabolites modulate the immune response by influencing T cell differentiation and Treg populations.

Dietary interventions, prebiotics, probiotics, and microbial metabolite supplementation, particularly SCFAs, have been proposed to restore gut microbial balance, reinforce epithelial barrier integrity, and attenuate inflammatory responses. However, the current clinical evidence remains heterogeneous as these effects are not consistently observed across studies. Despite substantial progress, significant gaps remain in our understanding of the precise mechanistic pathways linking gut dysbiosis, microbial metabolites, and skin inflammation in AD. The heterogeneity of AD further complicates the interpretation of the available data.
